# Involvement of Endoplasmic Reticulum Stress in Albuminuria Induced Inflammasome Activation in Renal Proximal Tubular Cells

**DOI:** 10.1371/journal.pone.0072344

**Published:** 2013-08-20

**Authors:** Li Fang, Da Xie, Xian Wu, Hongdi Cao, Weifang Su, Junwei Yang

**Affiliations:** Center for Kidney Disease, The Second Affiliated Hospital of Nanjing Medical University, Nanjing, Jiangsu Province, China; UCL Institute of Child Health, United Kingdom

## Abstract

Albuminuria contributes to the progression of tubulointerstitial fibrosis. Although it has been demonstrated that ongoing albuminuria leads to tubular injury manifested by the overexpression of numerous proinflammatory cytokines, the mechanism remains largely unknown. In this study, we found that the inflammasome activation which has been recognized as one of the cornerstones of intracellular surveillance system was associated with the severity of albuminuria in the renal biopsies specimens. In vitro, bovine serum albumin (BSA) could also induce the activation of NLRP3 inflammasome in the cultured kidney epithelial cells (NRK-52E). Since there was a significant overlap of NLRP3 with the ER marker calreticulin, the ER stress provoked by BSA seemed to play a crucial role in the activation of inflammasome. Here, we demonstrated that the chemical chaperone taurine-conjugated ursodeoxycholic acid (TUDCA) which was proved to be an enhancer for the adaptive capacity of ER could attenuate the inflammasome activation induced by albuminuria not only in vitro but also in diabetic nephropathy. Taken together, these data suggested that ER stress seemed to play an important role in albuminuria-induced inflammasome activation, elimination of ER stress via TUDCA might hold promise as a novel avenue for preventing inflammasome activation ameliorating kidney epithelial cells injury induced by albuminuria.

## Introduction

Proteinuria is a common feature of chronic kidney disease. However, the recognition that proteinuria is a possible contributor to the progression rather than a mere marker of the severity of glomerular damage represents a major change in the concepts of progressive kidney disease in recent years [1]. Accumulated evidences indicate that higher levels of proteinuria predict more rapid decline in renal function and more pronounced tubulointerstitial injury [2]–[4]. Progressive tubule injury and interstitial fibrosis, the major determinant in the progression of renal failure, has been proved to correlate with the magnitude of proteinuria [2], [4]. On the basis of these observations, it has been widely accepted that ultrafiltered proteins are toxin to the renal proximal tubule cells and can induce a cascade of biomolecular changes in renal tubular cells, which leads to the expression of numerous chemokines, adhesion molecules and proinflammatory cytokines [5]. This sterile inflammation has been found to be a universal response accompanying almost all kidney diseases and always correlates with worsening renal function [6]. However, the molecular mechanisms that regulate this inflammation in the progression of renal failure remain largely unknown.

Inflammasomes, the caspase-1-activating platforms, which are emerging as one of the cornerstones of the intracellular surveillance system, are activated upon cellular stress and then trigger the maturation of proinflammatory cytokines such as interleukin-1β and IL-18 to engage innate immune defenses [7]. Dysregulated inflammasome activation has been suggested to play pathogenic roles in a variety of complex diseases such as metabolic syndrome [8], [9], cancer [10] and infection [11]. Since inflammasome-dependent cytokines IL-1β and IL-18 have been proved to be critical mediators of tubulointerstitial inflammation [12]–[14], it seems that inflammasome activation could also participate in the process of tubulointerstitial fibrosis. Akosua Vilaysane et.al. reported that NLRP3 inflammasome activation, which was the most fully characterized inflammasome currently, played an important role in renal injury and could been identified as a possible therapeutic target in the treatment of patients with progressive CKD [15]. However, the science of inflammasome activation has only been explored recently in the field of nephrology and it is still unclear how this innate immune defense system involves in the various forms of kidney disease [16]. The objective of this study is to investigate whether the inflammasome is activated in proteinuric kidney diseases and the underlying mechanisms involved in inflammasome activation in albuminuria induced tubular injury in the progression of chronic kidney disease.

## Results

### The Inflammasome Activation is Associated with the Severity of Proteinuria

**Figure 1:**
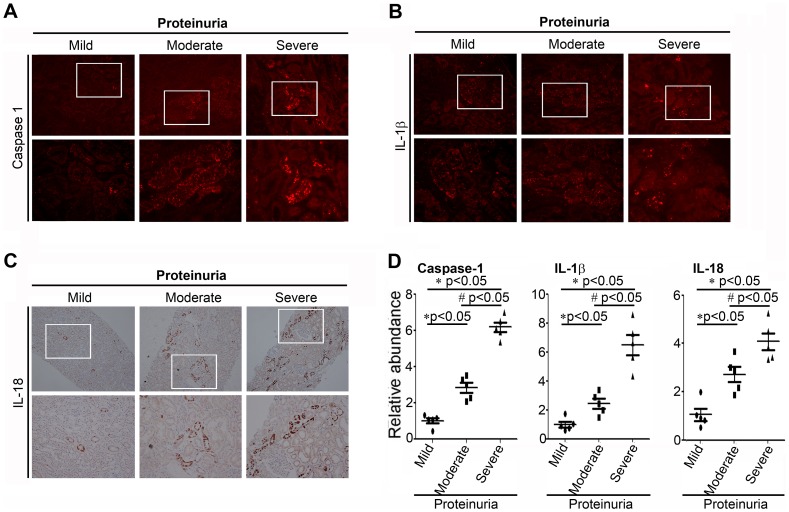
Expression of the inflammasome markers in renal tubular epithelia is associated with the severity of proteinuria. (The information and histological diagnoses are listed in Table S1). And samples were divided into the following categories according to the severity of proteinuria: mild (proteinuria<0.5 g/24 h); moderate (proteinuria 1.0–3.5 g/24 h); and severe (proteinuria>5 g/24 h). A. Representative micrographs by immunofluorescence staining of caspase-1 from patients with proteinuria, demonstrating the predominance of tubular staining for active caspase-1 and the correlation between intensity of tubular staining and proteinuria level. B. Representative micrographs by immunofluorescence staining of IL-1β. C. Representative micrographs by immunohistochemical staining of IL-18. D. Scatter diagram demonstrated the relative abundance of the semiquantitative histomorphometric analysis of the immunostaining of caspase-1, IL-1β, and IL-18 (n = 5). *P<0.05 vs. mild (proteinuria<0.5 g/24 h); # P<0.05 vs. moderate (proteinuria 1.0–3.5 g/24 h).

To determine whether the inflammasome was activated in proteinuric kidney disease, we first detected the intensity and distribution of caspase-1, IL-1β and IL-18 staining which were the markers of inflammasome activation in the human renal biopsies. In the renal biopsy specimen, the expressions of inflammasome related proteins such as caspase-1, IL-1β and IL-18 were not only observed in distal tubules but in proximal tubules. Staining of protocol biopsies showed constitutive IL-18 expression in the epithelium of distal tubules with the induction of immunoreactivity in the proteinuric patients where also proximal tubules were strongly positive. It suggested a correlation between the inflammasome activation in proximal tubules and the magnitude of proteinuria. As show in Figure 1A, B and C, in patients with mild proteinuria (less than <0.5 g/24 h), there was minimal tubular staining for caspase-1, IL-1β, and IL-18. In patients with moderate proteinuria (1∼ 3.5 g/24 h), there was much more obvious positive tubular staining for caspase-1, IL-1β, and IL-18, while the glomerular staining was relatively spared. However, in patients with severe proteinuria (>5 g/24 h), the tubular staining for caspase-1, IL-1β, and IL-18 was especially obvious. Shown in Figure 1D, semiquantitative histomorphometric analysis of the immunostaining further confirmed that the relative abundances of inflammasome markers caspase-1, IL-1β, and IL-18 were significantly higher in the severe proteinuric group than in the mild and moderate proteinuric groups. This observation indicated that the inflammasome activation characterized by the caspase-1 activation, IL-1β and IL-18 production were correlated with the severity of proteinuria.

### Activation of Inflammasome by Bovine Serum Albumin in NRK-52E Cells

**Figure 2:**
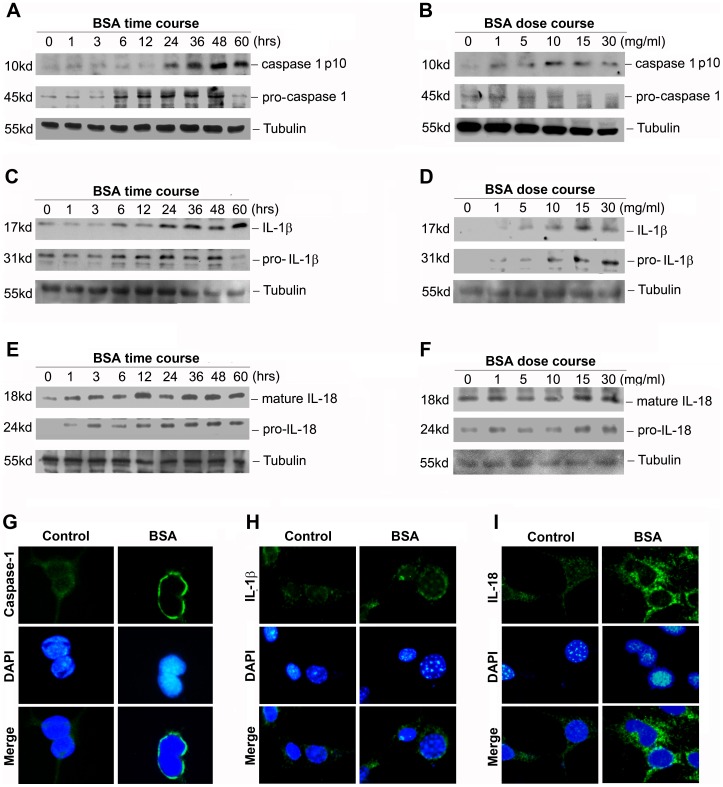
Bovine serum albumin induced the inflammasome activation in NRK-52E cells in vitro. A,C and E: Western blot analysis shows the caspase-1 activation and maturation of IL-1β and IL-18 protein in NRK-52E cells after treatment without or with 5 mg/ml BSA for various time periods in serum-free medium. The whole cell lysate was immunoblotted with caspase-1, IL-1β and IL-18 antibody, respectively. The same blot was reprobed with α-tubulin to confirm equal loading of each lane. B, D and F: Western blot analysis shows the caspase-1 activation and maturation of IL-1β and IL-18 protein in NRK-52E cells without or with different amounts of BSA for 12 h in serum-free medium. The whole cell lysate was also immunoblotted with caspase-1, IL-1β and IL-18 antibody, respectively. G through I: The inflammasome markers were detected by an indirect immunostaining in NRK-52E cells. NRK-52E cells were treated without (left column) or with 5 mg/ml BSA (right column) for 24 hours in serum-free medium. G: Immunostaining of caspase-1; H: Immunostaining of IL-1β; I: Immunostaining of IL-18.

**Figure 3:**
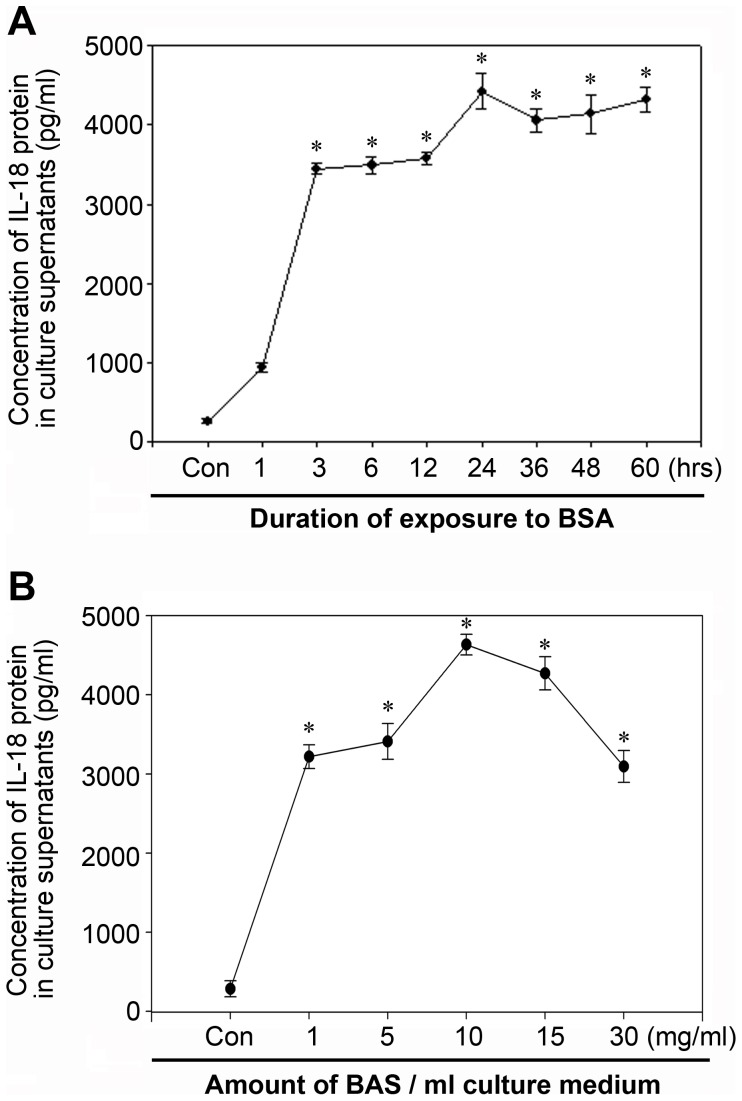
Bovine serum albumin induced up-regulated IL-18 secretion in supernatant of NRK-52E cells. A. Time course of IL-18 secretion in the supernatant of NRK-52E cells. Growth arrested NRK-52E cells were culture in DMEM-F12 containing 5 mg/ml BSA for 1–60 hours. IL-18 secretion in the culture was assayed by ELISA. B. Dose course of IL-18 secretion in the supernatant of NRK-52E cells. IL-18 secretion was dected as above. Data are presented as mean±SEM of three independent experiments. *P<0.05 vs. control.

Since the proximal tubule was the major site of reabsorption of filtrated albumin [17], we next used NRK-52E cell line as a vitro model to evaluate albuminuria’s role in regulating inflammasome activation. As shown in Figure 2 (A through F), following bovine serum albumin treatment, NRK-52E cells exhibited caspase-1 activation and maturation of IL-1β and IL-18 protein in a dose- and time-dependent manner, suggesting that bovine serum albumin could induce inflammasome activation in vitro. Graphical representation of the semi quantitative analysis of western blot results was presented in Figure S1. Concomitant with the western blot alterations, as shown by immunofluorescence staining in Figure 2 (G through I), it demonstrated that the caspase-1 antibody used had higher affinity for the active form of caspase-1, and the IL-1β and IL-18 proteins were much more obvious and located around nucleolus after BSA treatment. This was also confirmed by additional assay of IL-18 secretion in the culture supernatant of NRK-52E cells. As presented in Figure 3 (A and B), both time-response and dose-response studies revealed that BSA could up-regulate IL-18 secretion in the culture supernatants, with significant induction after a three-hour incubation with as little as 5 mg/ml albumin and beyond. However, IL-1β content in the culture supernatants was undetectable using the ELISA kit (data not shown).

### Bovine Serum Albumin Triggers NLRP3 Inflammasome Activation

**Figure 4:**
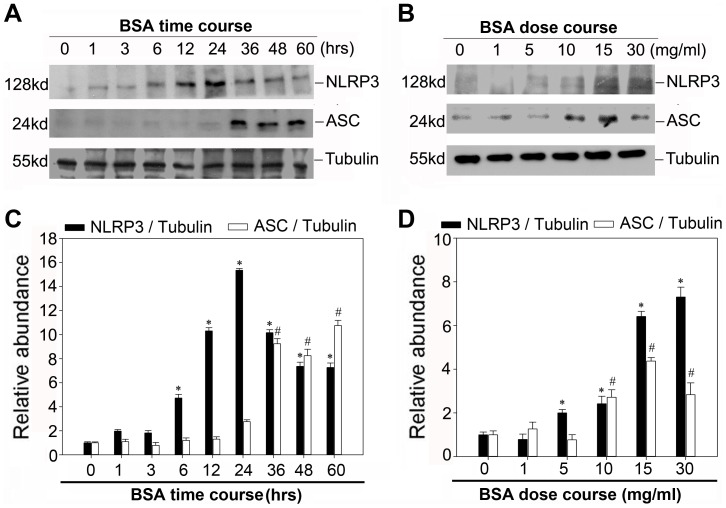
Bovine serum albumin induced NLRP3-inflammasome activation in NRK-52E cells. A. Western blot analysis showed the expression of NLRP3 and ASC proteins in NRK-52E cells after treatment without or with 5 mg/ml BSA for various time periods in serum-free medium. The whole cell lysate was immunoblotted with NLRP3 and ASC antibody, respectively. The same blot was reprobed with α-tubulin to confirm equal loading of each lane. B. Western blot analysis shows the expression of NLRP3 and ASC proteins in NRK-52E cells without or with different amounts of BSA for 12 h in serum-free medium. C and D: Graphical presentation shows the relative abundances of NLRP3 and ASC after normalization with α-tubulin. Data are presented as mean±SEM of three independent experiments. *P<0.05 vs. normal control (the relative abundance of NLRP3 protein level); # P<0.05 vs. normal control (the relative abundance of ASC protein level).

**Figure 5:**
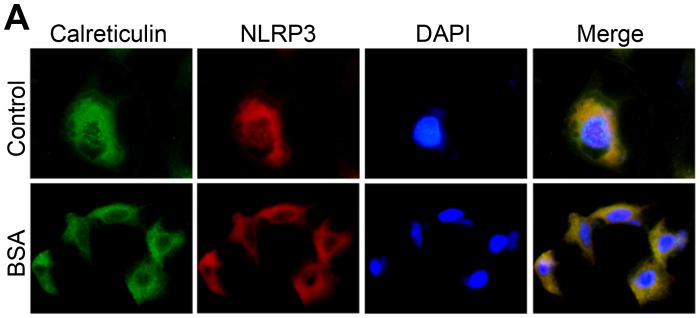
The NLRP3 protein overlaps with the ER marker calreticulin in NRK-52E cells. Growth arrested NRK-52E cells were treated without (upper line) or with 5 mg/ml BSA (under line) for 24 hours in serum-free medium. NLRP3 protein (red) and calreticulin protein (green) were detected by an indirect immunofluorescence staining.

Since NLRP3 inflammasome was the most fully characterized inflammasome currently, we next examined the expression of NLRP3 and the adaptor protein apoptosis-associated speck like protein (ASC) after treatment with BSA in vitro. As illustrated in Figure 4 A and B, western blot analysis revealed that BSA induced the expression of NLRP3 and ASC in both dose-dependent and time-dependent manner. NLRP3 protein expression increased as early as 6 hour while ASC protein expression protein increased after 36 hours incubation. Consistent with previous report [18], indirect immunofluorescence staining in Figure 5 shows a significant overlap of NLRP3 with the ER marker calreticulin under non-stimulatory conditions. However, after inflammasome stimulation with BSA, the pattern of this co-localization of NLRP3 and calreticulin altered significantly. NLRP3 relocated into the perinuclear space, but was still co-localized with calreticulin that stained positively for ER.

### Bovine Serum Albumin Induces Endoplasmic Reticulum Stress in NRK-52E Cells

**Figure 6:**
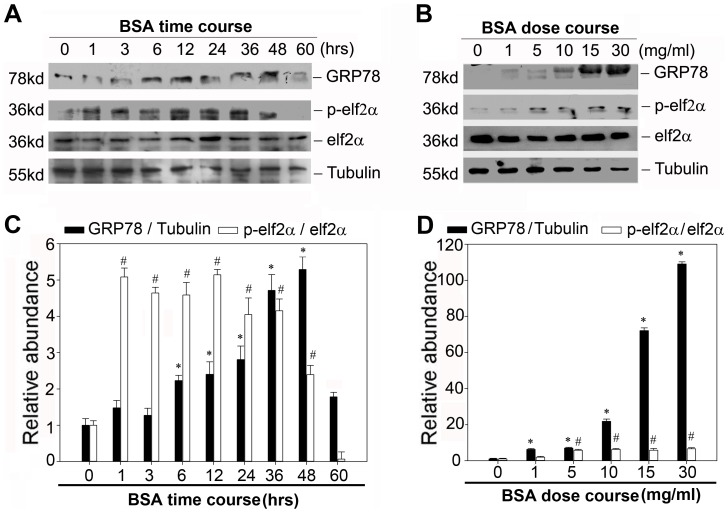
Bovine serum albumin induced endoplasmic reticulum stress in NRK-52E cells. A. Western blot analysis shows the expression of GRP78 and the phosphorylation of eIF2a in NRK-52E cells after treatment without or with 5 mg/ml BSA for various time periods in serum-free medium. The whole cell lysate was immunoblotted with GRP78, phosphorylated- eIF2α and eIF2α antibody, respectively. The same blot was reprobed with α-tubulin to confirm equal loading of each lane. B. Western blot analysis shows the expression of GRP78 and the phosphorylation of eIF2a in NRK-52E cells without or with different amounts of BSA for 12 h in serum-free medium. C and D: Graphical presentation shows the relative abundances of GRP78 after normalization with α-tubulin and the phosphorylation of eIF2α after normalization with eIF2α. Data are presented as mean±SEM of three independent experiments. *P<0.05 vs. normal control (the relative abundance of GRP78 protein level); # P<0.05 vs. normal control (the phosphorylation of eIF2α).

Since the previous results demonstrated strong evidence for the correlation of NLRP3 proteins and the endoplasmic reticulum structures, we speculated that the dynamics of ER might play a vital role in regulating the NLRP3 inflammasome activation. To test this hypothesis, we first examined the expression of GRP78 and the phosphorylation of eIF2α after BSA treatment. As shown in Figure 6, BSA markedly induced the expression of GRP78 and eIF2α phosphorylation in both dose-dependent and time-dependent manner. These data indicated that renal tubular cells exposed to bovine serum albumin suffered from ER stress.

### Reduction of ER Stress via Taurine-conjugated Derivative (TUDCA) Diminishes NLRP3 Inflammasome Activation

**Figure 7:**
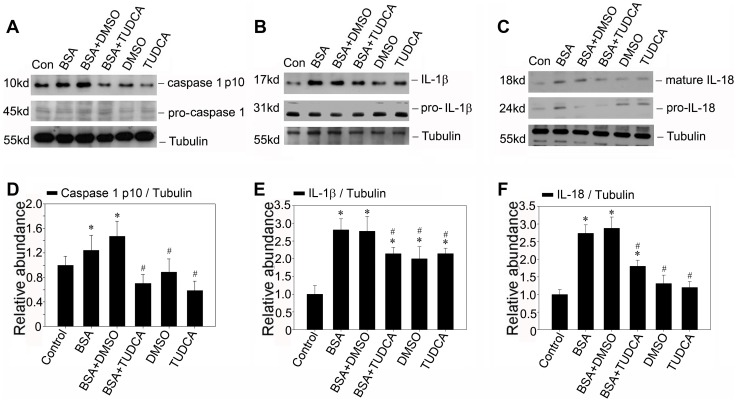
TUDCA attenuates the inflammasome activation induced by BSA in NRK-52E cells in vitro. A, B and C: Western blot analysis shows TUDCA attenuates the caspase-1 activation and maturation of IL-1β and IL-18 protein induced by BSA in NRK-52E cells. Growth arrested NRK-52E cells were pretreated without (same amount of DMSO) or with 100 umol/L TUDCA for 0.5 hours, and then followed by incubation without or with 5 mg/ml BSA for 12 hours as indicated. C, D and E: Graphic presentation showed the relative abundance of caspase-1, IL-1β and IL-18 protein after normalization with α-tubulin in various groups. Data are presented as mean±SEM of three independent experiments. *P<0.05 vs. normal control; # *P*<0.05 vs. group with BSA treatment.

To further demonstrate the significance of ER stress in the regulation of NLRP3 inflammasome activation, we used TUDCA a known chemical chaperone that enhanced the cytoprotective capacity of the ER [19]. We treated NRK-52E cells with BSA in the absence or presence of TUDCA. It was found that TUDCA treatment could repress BSA induced activation of caspase-1 as determined by immunoblotting for the active p10 subunit as shown in Figure 7A. Similarly, the maturation of IL-1β and IL-18 protein was also reduced after TUDCA pretreatment compared with the BSA treated group. These data further confirmed that the ER stress played an important role in the inflammasome activation. Hence, it suggested that TUDCA which regulated the dynamic of ER stress might abrogate the BSA induced inflammasome activation.

### The Inflammasome is Activated in the Renal Biopsy Specimens of Diabetic Patients

**Figure 8:**
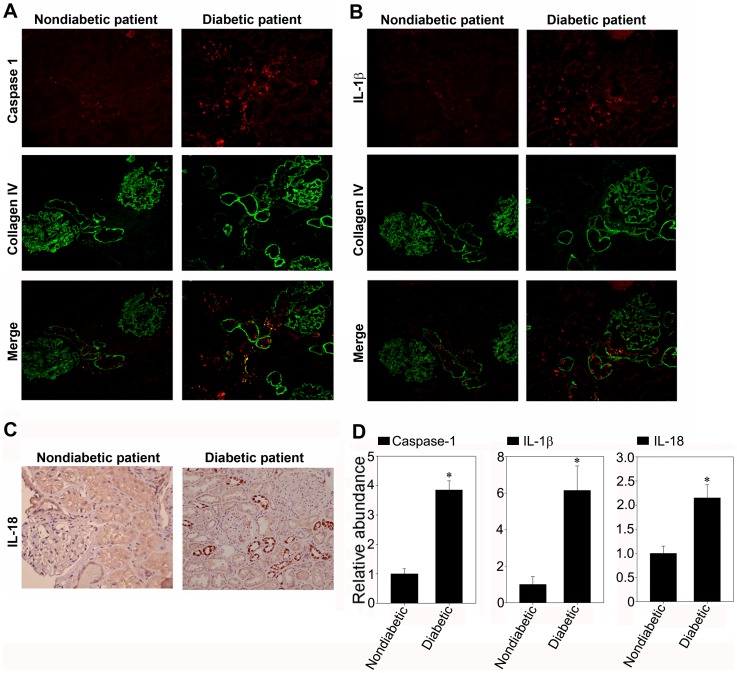
The inflammasome markers were activated in the renal tubular epithelia of diabetic nephropathy in the renal biopsies. A. Representative micrographs by immunofluorescence staining of caspase-1 (red) and collagen IV (green) in nondiabetic patients and diabetic patients, demonstrating the predominance of tubular staining for active caspase-1 in diabetic renal biopsies. B. Representative micrographs by immunofluorescence staining of IL-1β (red) and collagen IV (green). C. Representative micrographs by immunohistochemical staining of IL-18. D. The histogram demonstrated the relative abundance of the semiquantitative histomorphometric analysis of the immunostaining of caspase-1, IL-1β, and IL-18 (n = 5). *P<0.05 vs. the patients with mild mesangium proliferative glomerulonephritis.

Given the increasing prevalence of diabetes in both developed and developing countries, diabetic nephropathy has become the most common cause of proteinuric kidney disease. So we also investigated the intensity and distribution of caspase-1, IL-1β and IL-18 staining in the renal biopsy specimens of diabetic patients. As show in Figure 8, immunostaining sections showed strong staining of caspase-1, IL-1β and IL-18 in the renal tubule of diabetic patients whose proteinuria were about 5.21±1.863 g/24 h, as compared to the patients with mild mesangium proliferative glomerulonephritis whose proteinuria were about 0.32±0.174 g/24 h. This observation suggested that the inflammasome was activated in the renal tubule of diabetic nephropathy.

### TUDCA Ameliorates the Inflammasome Activation in Diabetic Kidney

**Figure 9:**
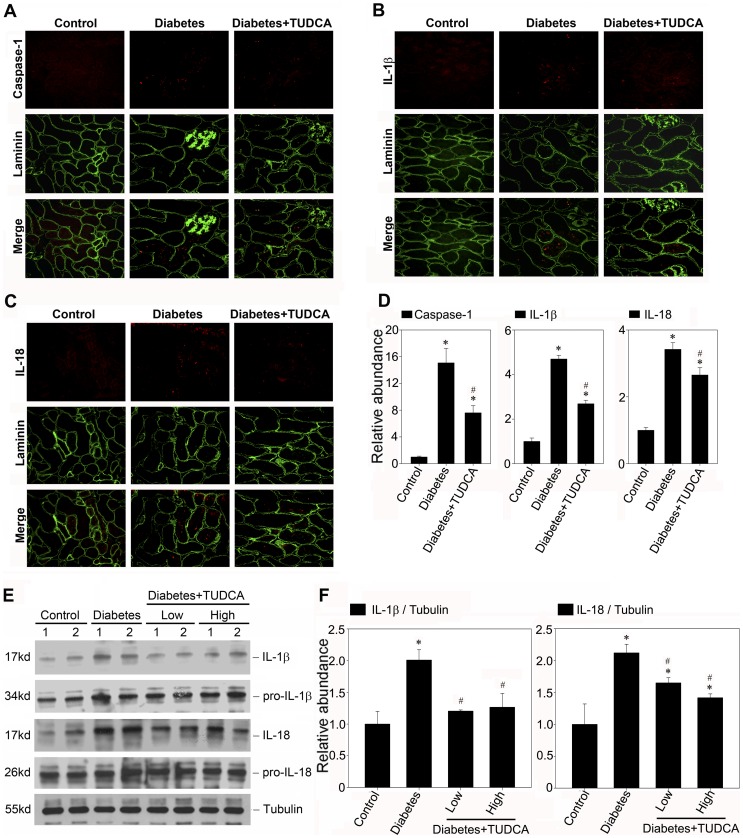
TUDCA attenuates the inflammasome activation in STZ induced diabetic nephropathy. A, B and C: Kidney cryosections were stained by an indirect immunofluorescence technique. Representative micrographs from different groups as indicated showed the immunostaining of inflammasome markers in diabetic nephropathy treated without (middle column) or with 500 mg/kg/day TUDCA (right column). D: The histogram demonstrated the relative abundance of the semiquantitative histomorphometric analysis of the immunostaining of caspase-1, IL-1β, and IL-18. E: Western blot analysis showed TUDCA suppressed the maturation of IL-1β and IL-18 protein in STZ induced diabetic nephropathy. The kidney lysate (made from the pool of kidneys from six animals/group) were separated on a SDS-polyacrylamide gel and immunoblotted with a specific monoclonal antibody against IL-1β, IL-18 and α-tubulin, respectively. Samples from two individual animals were used at each time point. F: Quantitative determination of IL-1β and IL-18 protein abundance after normalization with α-tubulin. Data are presented as means ± SEM of three experiments. n = 6, *P<0.05 vs. normal control. #*P*<0.05 vs. diabetic group.

As indicated above, we have demonstrated that TUDCA could be able to suppress the inflammasome activation in vitro; we next tested if, in vivo, TUDCA had the similar effect. It was also found that the caspase-1 activation in renal tubule of diabetic kidney was alleviated by the TUDCA treatment (Figure 9A). Concomitant with caspase-1 activation, as shown in Figure 9 (B through E), the cytokine IL-1β and IL-18 maturation which was observed in the diabetic kidneys over 28-day time course was also suppressed by TUDCA. These observations implied that the ER cytoprotective enhancer TUDCA could diminish the proteinuria induced inflammasome activation in the renal tubule and could be identified as a novel therapeutic agent for preventing the activation of inflammasome in proteinuric kidney diseases.

## Discussion

In recent decades, the progression from chronic kidney disease (CKD) to end stage renal disease has been a critical focus of research in nephrology and great effects have been made to develop strategies to halt or slow down the progression of chronic kidney disease. Proteinuria, the most common feature of chronic kidney disease, is an important target for renoprotection because it is a major risk factor for renal disease progression [20], [21]. Therapeutic strategies aimed at nonspecifically reducing proteinuria, such as tight blood pressure control, ACE inhibition, and possibly low-protein diets, are recommended. Growing evidences from the Ramipril Efficacy in Nephropathy (REIN) study [22], the Reduction of End Points in NIDDM with Angiotensin II antagonist Losartan (RENAAL) study [23] and others have showed that the level of proteinuria is a predictor of CKD progression, irrespective of blood pressure control and treatment randomization. The early recognition of the correlation between proteinuria and progression of CKD is that the magnitude of proteinuria is a reflection of the severity of the underlying glomerular disease. However, recently, it has also been suggested that proteinuria directly contributes to renal injury, in particular, to tubulointerstitial fibrosis, the final common histopathology features of all kidney diseases of any etiology [5], but the mechanism of proteinuria induced tubular injury is not fully understood.

Inflammasomes, the high-molecular weight, caspase-1-activating platforms, are emerging as key regulators of the innate immune response. Emerging literature has suggested that inflammasome might serve as a sophisticated system for sensing signals of “danger”, such as pathogenic microbes or host-derived signals of cellular stress [7]. IL-1β and IL-18 are important inflammasome effect molecules which require caspase-1-mediated cleavage for full activation and secretion. Although the concept of “inflammasome” was not specifically mentioned previously, mounting evidence has emphasized the importance of IL-1β and IL-18 in their ability to mediate tubulointerstitial fibrosis and tubular epithelial cell damage [12]–[14]. Previous investigations have showed that ultrafiltered proteins were toxic to the renal proximal tubule cells and could activate the expression of numerous chemokines, adhesion molecules and proinflammatory cytokines [24]. Accordingly, we have enough reasons to believe that the activation of inflammasome may be a critical inflammatory candidate in the progression of tubulointerstitial fibrosis. In this study, we indicated that kidney biopsies from nephritic subjects displayed stronger staining of inflammasome marker proteins such as caspase-1, IL-1β and IL-18 than those from minimally proteinuric subjects, suggesting that the inflammasome activation was associated with the severity of proteinuria. This association was further supported by the vitro studies that bovine serum albumin could induce the caspase-1 activation and the maturation of IL-1β and IL-18 protein in a time- and dose-dependent manner in vitro.

Although inflammation appeared to be an important pathogenic factor of proteinuria, the possible pathogenic role of albumin itself, however, is not fully elucidated. Zhou at al. had reported a significant overlap of NLRP3 with the endoplasmic reticulum marker calreticulin, suggesting that resting NLRP3 localizes to endoplasmic reticulum structures [18]. It is conceivable to speculate that the dynamics of ER plays a vital role in regulating the NLRP3 inflammasome activation. Indeed, we found that bovine serum albumin could up-regulate the expressions of NLRP3 protein and ASC protein, indicating the activation of NLRP3 inflammasome. And in accordance with the previous study [25], [26], our data also confirmed that renal tubular cells exposed to bovine serum albumin suffered from ER stress. To further confirm the role of ER stress in the NLRP3 inflammasome activation, we assessed the effect of chemical chaperone taurine-conjugated ursodeoxycholic acid (TUDCA), which has been proved to enhance the adaptive capacity of the ER [19]. TUDCA could attenuate the NLRP3 inflammasome activation not only in vitro but also in vivo. This investigation suggested that the bovine serum albumin induced NLRP3 inflammasome activation might be mediated by ER stress and reduction of ER stress via chemical chaperone might diminish NLRP3 inflammasome activation.

In summary, we demonstrated both *in vivo* and *in vitro* that severe proteinuria could induce the inflammasome activation in the renal tubule, the main contributor to the progressive tubulointerstitial fibrosis. Moreover, we also showed ER stress seemed to play an important role in BSA-induced inflammasome activation so that reduction of ER stress via chemical chaperone TUDCA may hold promise as novel avenues for impeding NLRP3 inflammasome activation and halting the progression of proteinuric kidney disease.

## Materials and Methods

### Ethics Statement

All of the following details of the study were approval by the responsible ethics committee of Nanjing Medical University (Permit Number: KY2012019). The written informed consent was supplied by the patients before the study.

The animal ethics approval was obtained from the Animal Care and Use Committee of Nanjing Medical University. All experiments were conducted in accordance with the Guide for the Care and Use of Laboratory Animals adopted by the Institutional Animal Care and Use Committee (IACUC) to ensure the proper care and welfare of animals involved in research. All the mice were housed in single cages and the housing room was maintained at a 12 h light/dark cycle (lights on at 8.00 h) at 22±3°C. Food and water were provided ad libitum. Mice were sacrificed under anesthesia. All the animal experiments were made to minimize suffering and reduce the number of animals used.

### Human Renal Biopsy Specimens

Renal biopsy samples were obtained from patients undergoing diagnostic evaluation at the Division of Nephrology, 2^nd^ Affiliated Hospital of Nanjing Medical University. A total of 15 subjects were (age range, 29 to 45) selected from our database with the criteria of having at least ten glomeruli in the block available for histological sectioning. All biopsy specimens were evaluated by a single pathologist who was unaware of the results of molecular studies (The information and histological diagnoses are listed in Table S1). Samples were divided into the following categories according to the severity of proteinuria: mild (proteinuria<0.5 g/24 h); moderate (proteinuria 1.0–3.5 g/24 h); and severe (proteinuria>5 g/24 h).

### Cell Culture

Rat renal proximal tubule epithelial cells (NRK-52E) were acquired from the Cell Resource Center of Shanghai Institutes for Biological Sciences, Chinese Academy of Sciences. Cells were cultured in DMEM-F12 supplemented with 10% newborn bovine serum (GIBCO, Grand Island, NY) [27]. For bovine serum albumin (BSA, A8806, Sigma, St. Louis, MO) treatment, NRK-52E cells were seeded at 80% confluence in complete medium containing 10% newborn bovine serum. Twenty-four hours later, the cells were changed to serum-free medium and incubated for additional 16 hours. Then BSA was added for various periods of time as scheduled. The cells were then collected for further characterization. The bovine serum albumin (A8806, Sigma-Aldrich) and the chemical chaperone taurine-conjugated ursodeoxycholic acid (TUDCA, T0266, Sigma -Aldrich) were obtained from Sigma (St. Louis, MO, USA).

### Animal Model

Male CD-1 mice weighed 18 to 22 grams were acquired from the Specific Pathogen-Free (SPF) Laboratory Animal Center of Nanjing Medical University. According to the guidelines of the Institutional Animal Care and Use Committee of the National Institutes of Health at Nanjing Medical University, all the animals were treated humanely and rendered diabetic by daily intraperitoneally injections of 50 mg/kg streptozotocin (STZ, Sigma, St. Louis, MO) for 5 days. For the sham-operated group, normal saline was administered. Tail blood glucose (TBG) levels were monitored consecutively and diabetic status was confirmed by the manifestation of weight loss, polyuria, and TBG level greater than 500 mg/dl. The diabetic mice were randomly assigned into three groups: diabetic mice to be treated with normal saline, diabetic mice to be treated with 250 mg/kg/day TUDCA and diabetic mice to be treated with 500 mg/kg/day TUDCA as previously described [19], [28]. To euthanize the mice, pentobarbital sodium (150–200 mg/kg, intraperitoneally) was used at 1 and 2- weeks after the treatment; serum and urine were collected and the kidneys were harvested.

### Immunostaining

Indirect immunofluorescence staining was performed according to an established procedure [29]. Briefly, cells cultured on cover slips were washed twice with cold PBS and fixed with cold methanol/acetone (1:1) for 10 min at -20°C. Following three extensive washings with PBS, the cells were blocked with 2% normal donkey serum in PBS buffer for 40 min at room temperature. Then the cells were incubated with anti-calreticulin (ab14234, Abcam) and anti-NALP3/anti-NLRP3 (ab109314, Abcam), followed by staining with FITC- or TRITC conjugated secondary antibodies. Cells were double stained with 4, 6-diamidino-2-phenylindole to visualize the nuclei. For immunostaining of kidney sections, cryo-sections at 5 micron thickness were prepared and fixed in cold methanol/acetone (1:1) for 10 min. After being blocked with 2% normal donkey serum in PBS for 40 min, the sections were incubated with primary antibodies against caspase1 (sc-514, Santa Cruz biotechnology), IL-1β (sc-7884, Santa Cruz biotechnology) and laminin (L9393, Sigma-Aldrich), respectively, in PBS containing 1% BSA overnight at 4°C. As a negative control, the primary antibody was replaced with either nonimmune mouse or rabbit IgG, corresponding to species of the primary antibodies. Sections were then washed thoroughly in PBS and incubated for 1 hour with fluorescein isothiocyanate-conjugated secondary antibody (Sigma-Aldrich) at a dilution of 1:500 in PBS containing 1% BSA. Slides were mounted with vectashied antifade mounting media (Vector Laboratories, Burlingame, CA) and viewed with a Nikon Eclipse 80i Epi-fluorescence microscope equipped with a digital camera (DS-Ri1, Nikon) [30]. In each experimental setting, images were captured with identical light exposure parameters and aperture settings. Immunohistochemical staining was performed by use of the Vector M.O.M. immunodetection kit (Vector Laboratories), as described previously [31]. The primary antibody used was anti-IL18 (sc-7954, Santa Cruz Biotechnology). As a negative control, the primary antibody was replaced with nonimmune normal IgG, and no staining occurred. Slides were also viewed with a Nikon Eclipse 80i Epi-fluorescence microscope equipped with a digital camera (DS-Ri1, Nikon).

### Western Blot Analysis

Cells were lysed with SDS sample buffer (62.5 mmol/l Tris·HCl, pH 6.8, 2% SDS, 10% glycerol, 50 mmol/l dithiothreitol, and 0.1% bromophenol blue). Kidney tissue was homogenized by a polytron homogenizer (Brinkmann Instruments, Westbury, NY), and the supernatant was collected after centrifugation at 13,000 *g* at 4°C for 20 min as described previously [31]. After protein concentration was determined using a bicinchoninic acid (BCA) protein assay kit (Sigma), the tissue lysate was mixed with an equal amount of 4X SDS sample buffer. Samples were heated at 100°C for 5–10 min before loading and separated on precast 10% SDS-polyacrylamide gels (Bio-Rad, Hercules, CA). The proteins were electro transferred to a nitrocellulose membrane (Amersham, Arlington Heights, IL) in transfer buffer containing 48 mmol/l Tris·HCl, 39 mmol/l glycine, 0.037% SDS, and 20% methanol at 4°C for 1 h. Nonspecific binding to the membrane was blocked for 1 hour at room temperature with 5% Carnation nonfat milk in TBS buffer (20 mmol/l Tris·HCl, 150 mmol/l NaCl, and 0.1% tween 20). The membranes were incubated for 16 hours at 4°C with various primary antibodies in TBS buffer containing 5% milk at the dilutions specified by the manufacturers. Binding of primary antibodies was followed by incubation for 1 hour at room temperature with the secondary horseradish peroxidase-conjugated IgG in 1% nonfat milk. The signals were visualized with the enhanced chemiluminescence system (ECL, Amersham), as described previously [27].

### Assay of IL-18 Protein in Culture Supernatants

NRK-52E cells were grown to confluence in six well cell culture plates. The growth was arrested and exposed to bovine serum albumin (1–30 mg/ml) for pre-defined time periods (1–60 hours ) at 37°C. Supernatants were collected and detection of IL-18 was performed using an IL-18 ELISA kit. Briefly, micro wells were coated (100 µl/well) with diluted capture antibody. After overnight incubation, washing, blocking, and repeated washing, 100 µl of the standards or samples was added to each well for 2 hours at room temperature. The wells were washed, and 100 µl of diluted detection antibody and 100 µl of diluted enzyme reagent were added to each well for 1 hour at room temperature. The wells were washed again and then 100 µl of substrate solution was added to each well for 30 minutes at room temperature in the dark. Finally, 50 µl of stop solution was added to each well, and extinction was measured within 30 minutes, using a microplate reader (Elx800, BIO-TEK) set to 450 nm.

### Statistical Analysis

All data examined were expressed as means ± SE. Each experiment was repeated three times independently. For the immunostaining, semiquantitative histomorphometric analysis was performed by using Image-Pro plus 6.0 software. For western blot analysis, quantitation was performed by scanning and analyzing the intensity of the hybridization signals using NIH Imagine software. Statistical analysis was performed using Sigma Stat Software (Jandel Scientific Software, San Rafael, CA). Comparisons between groups were made using one-way ANOVA, followed by a *t-*test. A “*P* value*<*0.05″ was considered significant.

## Supporting Information

Figure S1 **Semi-quantitative analysis of BSA induced inflammasome activation in NRK-52E cells.** Graphic presentation showed the relative abundance of caspase-1, IL-1β and IL-18 protein after normalization with α-tubulin in various groups. Data are presented as mean±SEM of three independent experiments. A. Time course; B. Dose course. * *P*<0.05 vs. normal control (the relative abundance of caspase-1 p10 protein level); #*P*<0.05 vs. normal control (the relative abundance of mature IL-1β protein level); §*P*<0.05 vs. normal control (the relative abundance of mature IL-18 protein level).
(TIF)

Table S1 **The information and histological diagnoses of selected patients.**
(DOC)

## References

[1] Zandi-Nejad K, Eddy AA, Glassock RJ, Brenner BM (2004) Why is proteinuria an ominous biomarker of progressive kidney disease? Kidney Int Suppl: S76–89. 10.1111/j.1523-1755.2004.09220.x 15485426

[2] Burton C, Harris KP (1996) The role of proteinuria in the progression of chronic renal failure. Am J Kidney Dis 27: 765–775. 8651239 10.1016/s0272-6386(96)90512-0

[3] Remuzzi G, Bertani T (1998) Pathophysiology of progressive nephropathies. N Engl J Med 339: 1448–1456. 9811921 10.1056/NEJM199811123392007

[4] Eddy AA (2004) Proteinuria and interstitial injury. Nephrol Dial Transplant 19: 277–281. 14736944 10.1093/ndt/gfg533

[5] Zoja C, Morigi M, Remuzzi G (2003) Proteinuria and phenotypic change of proximal tubular cells. J Am Soc Nephrol 14 Suppl 1S36–41. 12761237 10.1097/01.asn.0000068626.23485.e0

[6] Zeisberg M, Neilson EG (2010) Mechanisms of tubulointerstitial fibrosis. J Am Soc Nephrol 21: 1819–1834. 20864689 10.1681/ASN.2010080793

[7] Schroder K, Tschopp J (2010) The inflammasomes. Cell 140: 821–832. 20303873 10.1016/j.cell.2010.01.040

[8] Stienstra R, van Diepen JA, Tack CJ, Zaki MH, van de Veerdonk FL, et al (2011) Inflammasome is a central player in the induction of obesity and insulin resistance. Proc Natl Acad Sci U S A 108: 15324–15329. 21876127 10.1073/pnas.1100255108 PMC3174591

[9] Schroder K, Zhou R, Tschopp J (2010) The NLRP3 inflammasome: a sensor for metabolic danger? Science 327: 296–300. 20075245 10.1126/science.1184003

[10] Zitvogel L, Kepp O, Galluzzi L, Kroemer G (2012) Inflammasomes in carcinogenesis and anticancer immune responses. Nat Immunol 13: 343–351. 22430787 10.1038/ni.2224

[11] Dunne A (2011) Inflammasome activation: from inflammatory disease to infection. Biochem Soc Trans 39: 669–673. 21428959 10.1042/BST0390669

[12] Wetmore JB, Mahnken JD, Rigler SK, Ellerbeck EF, Mukhopadhyay P, et al (2013) Impact of race on cumulative exposure to antihypertensive medications in dialysis. Am J Hypertens 26: 234–242. 23382408 10.1093/ajh/hps019 PMC3626037

[13] Tesch GH, Yang N, Yu H, Lan HY, Foti R, et al (1997) Intrinsic renal cells are the major source of interleukin-1 beta synthesis in normal and diseased rat kidney. Nephrol Dial Transplant 12: 1109–1115. 9198037 10.1093/ndt/12.6.1109

[14] Bani-Hani AH, Leslie JA, Asanuma H, Dinarello CA, Campbell MT, et al (2009) IL-18 neutralization ameliorates obstruction-induced epithelial-mesenchymal transition and renal fibrosis. Kidney Int 76: 500–511. 19536084 10.1038/ki.2009.216

[15] Vilaysane A, Chun J, Seamone ME, Wang W, Chin R, et al (2010) The NLRP3 inflammasome promotes renal inflammation and contributes to CKD. J Am Soc Nephrol 21: 1732–1744. 20688930 10.1681/ASN.2010020143 PMC3013544

[16] Anders HJ, Muruve DA (2011) The inflammasomes in kidney disease. J Am Soc Nephrol 22: 1007–1018. 21566058 10.1681/ASN.2010080798

[17] Tojo A, Kinugasa S (2012) Mechanisms of glomerular albumin filtration and tubular reabsorption. Int J Nephrol 2012: 481520. 22685655 10.1155/2012/481520 PMC3363986

[18] Zhou R, Yazdi AS, Menu P, Tschopp J (2011) A role for mitochondria in NLRP3 inflammasome activation. Nature 469: 221–225. 21124315 10.1038/nature09663

[19] Ozcan U, Yilmaz E, Ozcan L, Furuhashi M, Vaillancourt E, et al (2006) Chemical chaperones reduce ER stress and restore glucose homeostasis in a mouse model of type 2 diabetes. Science 313: 1137–1140. 16931765 10.1126/science.1128294 PMC4741373

[20] Bakris GL (2008) Slowing nephropathy progression: focus on proteinuria reduction. Clin J Am Soc Nephrol 3 Suppl 1S3–10. 18178794 10.2215/CJN.03250807 PMC3152266

[21] de Zeeuw D, Remuzzi G, Parving HH, Keane WF, Zhang Z, et al (2004) Proteinuria, a target for renoprotection in patients with type 2 diabetic nephropathy: lessons from RENAAL. Kidney Int 65: 2309–2320. 15149345 10.1111/j.1523-1755.2004.00653.x

[22] Ruggenenti P, Perna A, Gherardi G, Garini G, Zoccali C, et al (1999) Renoprotective properties of ACE-inhibition in non-diabetic nephropathies with non-nephrotic proteinuria. Lancet 354: 359–364. 10437863 10.1016/S0140-6736(98)10363-X

[23] Eijkelkamp WB, Zhang Z, Remuzzi G, Parving HH, Cooper ME, et al (2007) Albuminuria is a target for renoprotective therapy independent from blood pressure in patients with type 2 diabetic nephropathy: post hoc analysis from the Reduction of Endpoints in NIDDM with the Angiotensin II Antagonist Losartan (RENAAL) trial. J Am Soc Nephrol 18: 1540–1546. 17409317 10.1681/ASN.2006050445

[24] Liu Y (2011) Cellular and molecular mechanisms of renal fibrosis. Nat Rev Nephrol 7: 684–696. 22009250 10.1038/nrneph.2011.149 PMC4520424

[25] Ohse T, Inagi R, Tanaka T, Ota T, Miyata T, et al (2006) Albumin induces endoplasmic reticulum stress and apoptosis in renal proximal tubular cells. Kidney Int 70: 1447–1455. 16955111 10.1038/sj.ki.5001704

[26] Lee YJ, Suh HN, Han HJ (2009) Effect of BSA-induced ER stress on SGLT protein expression levels and alpha-MG uptake in renal proximal tubule cells. Am J Physiol Renal Physiol 296: F1405–1416. 19211687 10.1152/ajprenal.90652.2008

[27] Yang Z, Xiaohua W, Lei J, Ruoyun T, Mingxia X, et al (2010) Uric acid increases fibronectin synthesis through upregulation of lysyl oxidase expression in rat renal tubular epithelial cells. Am J Physiol Renal Physiol 299: F336–346. 20484295 10.1152/ajprenal.00053.2010

[28] Fang L, Zhou Y, Cao H, Wen P, Jiang L, et al (2013) Autophagy Attenuates Diabetic Glomerular Damage through Protection of Hyperglycemia-Induced Podocyte Injury. PLoS One 8: e60546. 23593240 10.1371/journal.pone.0060546 PMC3623813

[29] Yang J, Liu Y (2002) Blockage of tubular epithelial to myofibroblast transition by hepatocyte growth factor prevents renal interstitial fibrosis. J Am Soc Nephrol 13: 96–107. 11752026 10.1681/ASN.V13196

[30] Yang J, Liu Y (2001) Dissection of key events in tubular epithelial to myofibroblast transition and its implications in renal interstitial fibrosis. Am J Pathol 159: 1465–1475. 11583974 10.1016/S0002-9440(10)62533-3 PMC1850509

[31] Wang X, Zhou Y, Tan R, Xiong M, He W, et al (2010) Mice lacking the matrix metalloproteinase-9 gene reduce renal interstitial fibrosis in obstructive nephropathy. Am J Physiol Renal Physiol 299: F973–982. 20844022 10.1152/ajprenal.00216.2010

