# Supplementary figures and images for: Involvement of Endoplasmic Reticulum Stress in Albuminuria Induced Inflammasome Activation in Renal Proximal Tubular Cells
Source: PLoS One. 2013 Aug 20;8(8):e72344. doi: 10.1371/journal.pone.0072344 (PMC3748031; doi:10.1371/journal.pone.0072344)

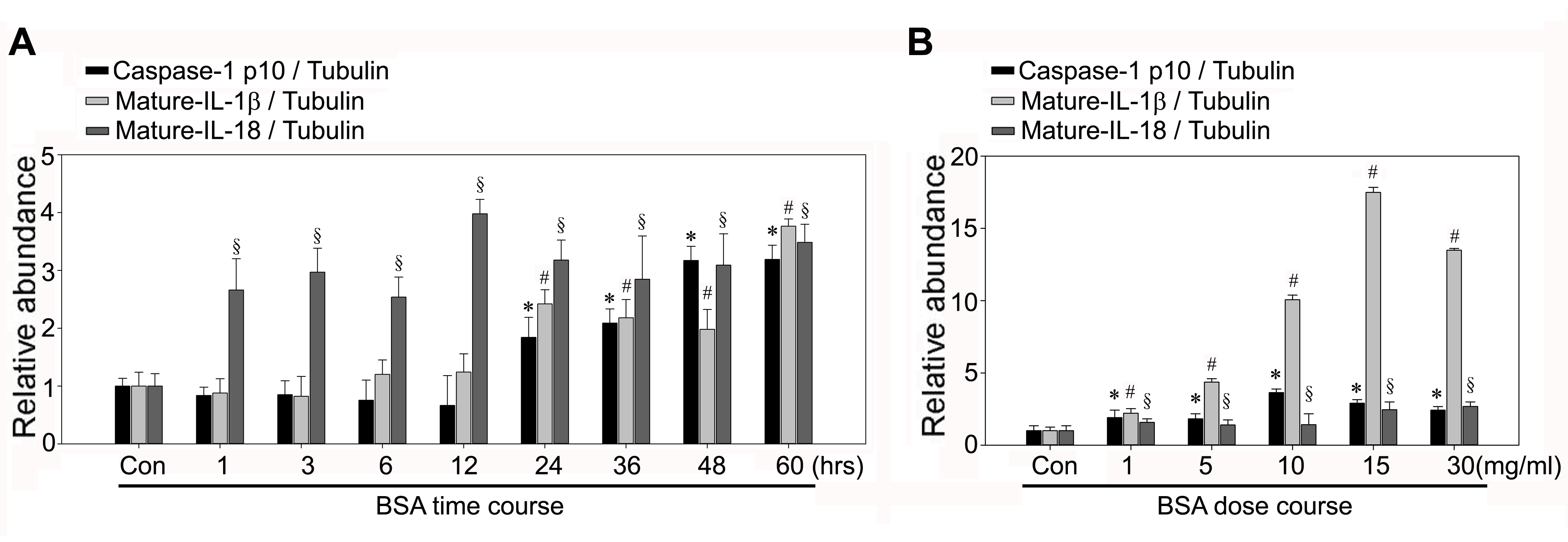

Supplement: Figure S1 — Semi-quantitative analysis of BSA induced inflammasome activation in NRK-52E cells. Graphic presentation showed the relative abundance of caspase-1, IL-1β and IL-18 protein after normalization with α-tubulin in various groups. Data are presented as mean±SEM of three independent experiments. A. Time course; B. Dose course. * P<0.05 vs. normal control (the relative abundance of caspase-1 p10 protein level); #P<0.05 vs. normal control (the relative abundance of mature IL-1β protein level); §P<0.05 vs. normal control (the relative abundance of mature IL-18 protein level). (TIF) [file pone.0072344.s001.tif]
