# Supplementary material for: Involvement of Endoplasmic Reticulum Stress in Albuminuria Induced Inflammasome Activation in Renal Proximal Tubular Cells
Source: PLoS One. 2013 Aug 20;8(8):e72344. doi: 10.1371/journal.pone.0072344 (PMC3748031; doi:10.1371/journal.pone.0072344)
Supplement: Table S1 — The information and histological diagnoses of selected patients. (DOC) [file pone.0072344.s002.doc]

**Table S1**

| **Number** | **Gender** | **Age** | **Proteinuria level** | **Histologic diagnosis** |
| --- | --- | --- | --- | --- |
| 1 | Male | 32 | 0.32g/24h | IgA nephropathy |
| 2 | Male | 35 | 0.35g/24h | IgA nephropathy |
| 3 | Female | 31 | 0.18g/24h | Mild mesangial proliferative glomerulonephritis |
| 4 | Female | 43 | 0.24g/24h | Mild mesangial proliferative glomerulonephritis |
| 5 | Male | 26 | 0.25g/24h | IgA nephropathy |
| 6 | Female | 49 | 1.55g/24h | Membranoproliferative glomerulonephritis |
| 7 | Male | 45 | 2.54g/24h | IgA nephropathy |
| 8 | Male | 34 | 3.25g/24h | IgA nephropathy |
| 9 | Female | 51 | 2.67g/24h | IgA nephropathy |
| 10 | Male | 50 | 3.13g/24h | Focal Segmental Glomerulosclerosis |
| 11 | Male | 42 | 5.87g/24h | Minimal Change Disease |
| 12 | Male | 33 | 8.45g/24h | Membranous glomerulonephritis |
| 13 | Female | 36 | 5.54g/24h | Membranous glomerulonephritis |
| 14 | Female | 53 | 9.15g/24h | Minimal Change Disease |
| 15 | Female | 48 | 10.23g/24h | Minimal Change Disease |
